# Identification of a Seven-lncRNA Immune Risk Signature and Construction of a Predictive Nomogram for Lung Adenocarcinoma

**DOI:** 10.1155/2020/7929132

**Published:** 2020-05-21

**Authors:** Donghui Jin, Yuxuan Song, Yuan Chen, Peng Zhang

**Affiliations:** ^1^Cardiovascular Thoracic Surgery Department, Tianjin Medical University General Hospital, 154 Anshan Road, Heping District, Tianjin, China; ^2^Department of Urology, Tianjin Medical University General Hospital, 154 Anshan Road, Heping District, Tianjin, China

## Abstract

**Background:**

The incidence of lung cancer is the highest of all cancers, and it has the highest death rate. Lung adenocarcinoma (LUAD) is a major type of lung cancer. This study is aimed at identifying the prognostic value of immune-related long noncoding RNAs (lncRNAs) in LUAD.

**Materials and Methods:**

Gene expression profiles and the corresponding clinicopathological features of LUAD patients were obtained from The Cancer Genome Atlas (TCGA). The least absolute shrinkage and selection operator (LASSO) Cox regression algorithm was performed on the prognostic immune-related lncRNAs to calculate the risk scores, and a risk signature was constructed. Survival analysis was performed to assess the prognostic value of the risk signature. A nomogram was also constructed based on the clinicopathological features and risk signature.

**Results:**

A total of 437 LUAD patients with gene expression data and clinicopathological features were obtained in this study, which was considered the combination set. They were randomly and equally divided into a training set and a validation set. Seven immune-related lncRNAs (AC092794.1, AL034397.3, AC069023.1, AP000695.1, AC091057.1, HLA-DQB1-AS1, and HSPC324) were identified and used to construct a risk signature. The patients were divided into the low- and high-risk groups based on the median risk score of -0.04074. Survival analysis suggested that patients in the low-risk group had a longer overall survival (OS) than those in the high-risk group (*p* = 1.478*e* − 02). A nomogram was built that could predict the 1-, 3-, and 5-year survival rates of LUAD patients (C-index of the nomogram was 0.755, and the AUCs for the 1-, 3-, and 5-year survivals were 0.826, 0.719, and 0.724, respectively). The validation and combination sets confirmed these results.

**Conclusion:**

Our study identified seven novel immune-related lncRNAs and generated a risk signature, as well as a nomogram, that could predict the prognosis of LUAD patients.

## 1. Introduction

Lung cancer has the highest incidence of all cancers and the highest death rate. It is more common in women than men (1). There are two main types of lung cancer, small cell lung cancer (SCLC, 15% of the cases) and non-small-cell lung cancer (NSCLC, 85% of the cases), which mainly includes lung adenocarcinoma (LUAD) and lung squamous cell carcinoma (LUSC) (2). Over the past decade, the availability of predictive biomarkers has led to a shift toward molecularly targeted therapies for NSCLC, particularly LUAD, which benefit many patients, especially patients with advanced or metastatic NSCLC. Specific treatments depend on biomarker status, such as the presence or absence of EGFR-activating mutations and ALK/ROS1 translocations. However, not every advanced patient has targetable mutations (3, 4).

The importance of the immune status in the tumor microenvironment (TME) has been gradually recognized. Dysfunction of the immune status in the TME is an important feature of tumors. In the course of tumor development, the immune system plays a dynamic role in cancer immune editing, which includes an elimination phase, an equilibrium phase, and an escape phase (5). Currently, immune checkpoint inhibitors, such as programmed death-1 (PD-1)/programmed death-ligand 1 (PD-L1), have become another main treatment for advanced NCSLC, especially in patients without targetable mutations (6). Therefore, it is necessary to identify more immune-related factors to promote the development of anticancer immunotherapy.

The human genome transcribes less than 2% of the protein-coding genes, and 85% of it is composed of noncoding RNAs, including long noncoding RNAs (lncRNAs) (7). lncRNAs account for a large part of the human genome and were once considered insignificant “noise” in the genome's repertoire of non-protein-coding transcripts (8). Recent studies have revealed the roles of lncRNAs in many biological processes, including transcriptional regulation and cell differentiation (9, 10). Furthermore, lncRNAs play vital roles in cancer immunity, such as antigen releasing and presentation, immune activation, immune cell migration, infiltration into cancer tissues, and cancer cell killing (5). However, the roles of immune-related lncRNAs in LUAD are still unclear. This study is aimed at exploring immune-related lncRNAs as biomarkers, as well as their prognostic roles in LUAD, by the integrated analyses of gene expression profiles.

## 2. Materials and Methods

### 2.1. Data Collection

We downloaded gene expression profiles and the corresponding clinicopathological features of the LUAD patients from The Cancer Genome Atlas (TCGA) (https://portal.gdc.cancer.gov/). We excluded samples from patients with survivals of ≤30 days because these patients might have died from nonneoplastic causes (11). The clinicopathological features included survival time, survival status, age, gender, and Union for International Cancer Control (UICC) stage (TNM stage). To increase the reliability of our research, we randomly and equally divided the entire dataset into a training set and a validation set and the whole dataset was considered a combination set.

### 2.2. Identification of Immune-Related lncRNAs

We extracted lncRNA expression data from the mRNA expression data according to the GENCODE project (http://www.gencodegenes.org) (12, 13). Based on the keywords IMMUNE_RESPONSE (Immune response M19817) and IMMUNE_SYSTEM_PROCESS (Immune system process M13664), immune-related genes were obtained from the Molecular Signatures Database (http://www.broadinstitute.org/gsea/msigdb/index.jsp) (14, 15). Next, Pearson's correlation analysis was performed between immune-related genes and lncRNA expression levels in samples to identify immune-related lncRNAs according to the correlation coefficients and *p* values (∣correlation coefficient | >0.6 and *p* < 0.001). To determine the prognostic value of immune-related lncRNAs, we conducted univariate Cox regression analysis on the immune-related lncRNAs in the training set by using the “survival” package in R (v3.6.1), and the hazard ratios (HRs) with 95% confidence intervals (CIs) were examined. *p* < 0.05 indicated that immune-related lncRNAs were correlated with overall survival (OS) and considered prognostic immune-related lncRNAs. Immune-related lncRNAs with HRs > 1 were considered to be risk factors, whereas HRs < 1 were considered protective factors.

### 2.3. Construction of the Risk Signature

A risk signature was constructed by performing the least absolute shrinkage and selection operator (LASSO) Cox regression algorithm on the prognostic immune-related lncRNAs using the “glmnet” and “survival” packages in R. The risk score for the signature was calculated using the following formula (16–18):

(1) $$\begin{array}{c} \text{Risk score}=\overset{n}{\underset{i=1}{\sum }}{\text{coef}}_{i}\times x_{i}, \end{array}$$

where coef_*i*_ is the coefficient and x_*i*_ is the expression of each prognostic immune-related lncRNA in each sample. The LUAD patients were divided into high-risk and low-risk groups based on the median risk score. Samples with risk scores no higher than the median risk score were assigned to the low-risk group; otherwise, they were assigned to the high-risk group. We next used the “survival” and “survminer” packages to construct a Kaplan–Meier survival curve to reveal the OS of the high-risk and low-risk groups. Log-rank *p* < 0.05 indicated a difference. We used the area under the curve (AUC) in the receiver operating characteristic (ROC) built by the “survivalROC” package to investigate the time-dependent prognostic value of the risk signature. Principal component analysis (PCA) was performed by the “limma” package to study the expression patterns in the different groups. What is more, we performed Estimation of STromal and Immune cells in MAlignant Tumor tissues using Expression data (ESTIMATE) analysis by using “estimate” package to compare the immune scores between the high-risk and low-risk groups to prove the difference in immunity.

### 2.4. Independence of the Risk Signature and Clinicopathological Features in the Prognostic Value

To investigate whether the risk signature and clinicopathological features were independent prognostic factors, we performed univariate and multivariate Cox regression analyses for each variable. Variables with *p* < 0.05 in both analyses indicated that they were independent prognostic factors.

### 2.5. Gene Set Enrichment Analysis

To reveal the potential function of the high-risk and low-risk groups, we performed Gene Set Enrichment Analysis (GSEA). *p* < 0.05 and a false discovery rate (FDR) of *q* < 0.25 indicated significant functional enrichment.

### 2.6. Building and Validation of a Nomogram

A nomogram is a statistical model of prognosis presented as a simple graph (19, 20). In the nomogram, each sample is assigned a point for each of its variables and the resulting total score predicts 1-, 3-, and 5-year survival rates (21). We used independent prognostic factors to build a nomogram using the “rms” package. A calibration plot (by a bootstrap method with 500 resamples) was used to validate the nomogram and concordance index (C-index), and time-dependent ROC curves were used to evaluate the discrimination of the nomogram.

### 2.7. Statistical Analysis

We used the chi-squared test and Fisher's exact test or Student's *t*-test to investigate differences in age, gender, TNM stage, and survival status between the high-risk and low-risk groups. *p* < 0.05 indicated significant difference.

## 3. Results

### 3.1. Data Acquisition

**Figure 1:**
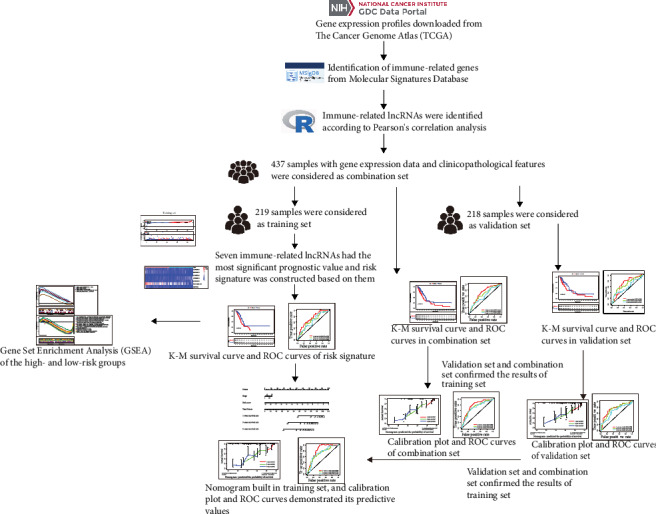
Flowchart of our study. Gene expression profiles were downloaded from The Cancer Genome Atlas (TCGA) database. Immune-related genes were extracted from the Molecular Signatures Database. Immune-related lncRNAs were identified according to Pearson's correlation. The training set was used to identify immune-related prognostic lncRNAs and establish a risk signature based on the immune-related prognostic lncRNAs. The prognosis analysis was validated by the validation set and the combination set, respectively. A nomogram was constructed by including the immune-related lncRNA signature and other prognosis-related clinical features in the training set and confirmed by the validation set and the combination set, respectively. Functional enrichment analyses based on the training set were utilized to explore immune-related functions. ROC: receiver operating characteristic.

A total of 437 LUAD patients with gene expression data and survival time (>30 days), survival status, age, gender, and TNM stage information were obtained from TCGA.  Figure 1 shows a flowchart of the steps involved in the study. Three hundred thirty-two immune-related genes were included from the Molecular Signatures Database. Based on ∣correlation coefficient | >0.6 and *p* < 0.001, 429 immune-related lncRNAs were identified. Four hundred thirty-seven patients were considered to be a combination set, which was randomly and equally divided into a training set (219 patients) and a validation set (218 patients) (Table 1). There were no differences in clinicopathological features between the two sets.

### 3.2. Construction of the Risk Signature

**Figure 2:**
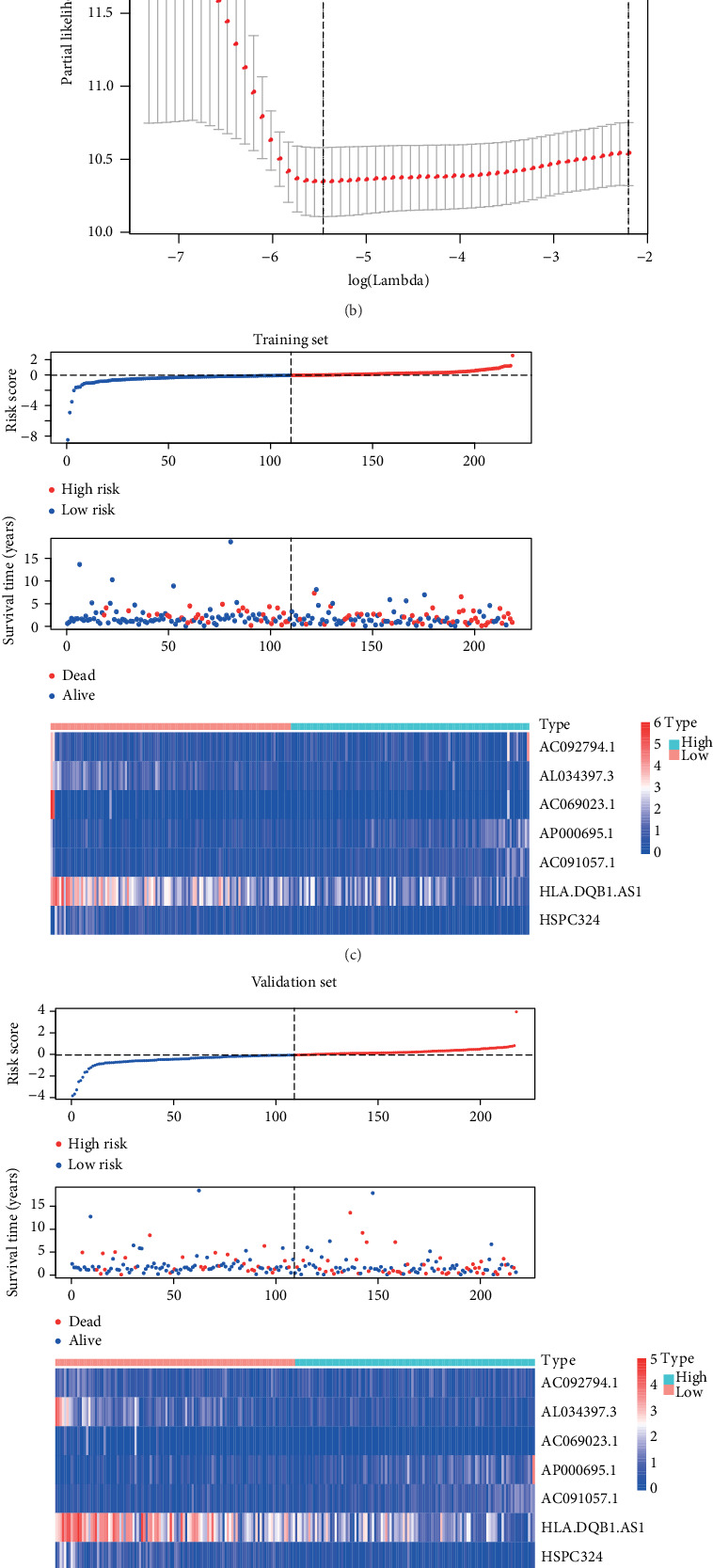
(a, b) The figures of LASSO coefficient distribution and partial likelihood deviation of the LASSO coefficient distribution of the training set. (c–e) Risk plots of the three sets. In each set, the risk score distribution, patients' survival status, and gene expression in the low- and high-risk groups were displayed. LASSO: least absolute shrinkage and selection operator.

**Figure 3:**
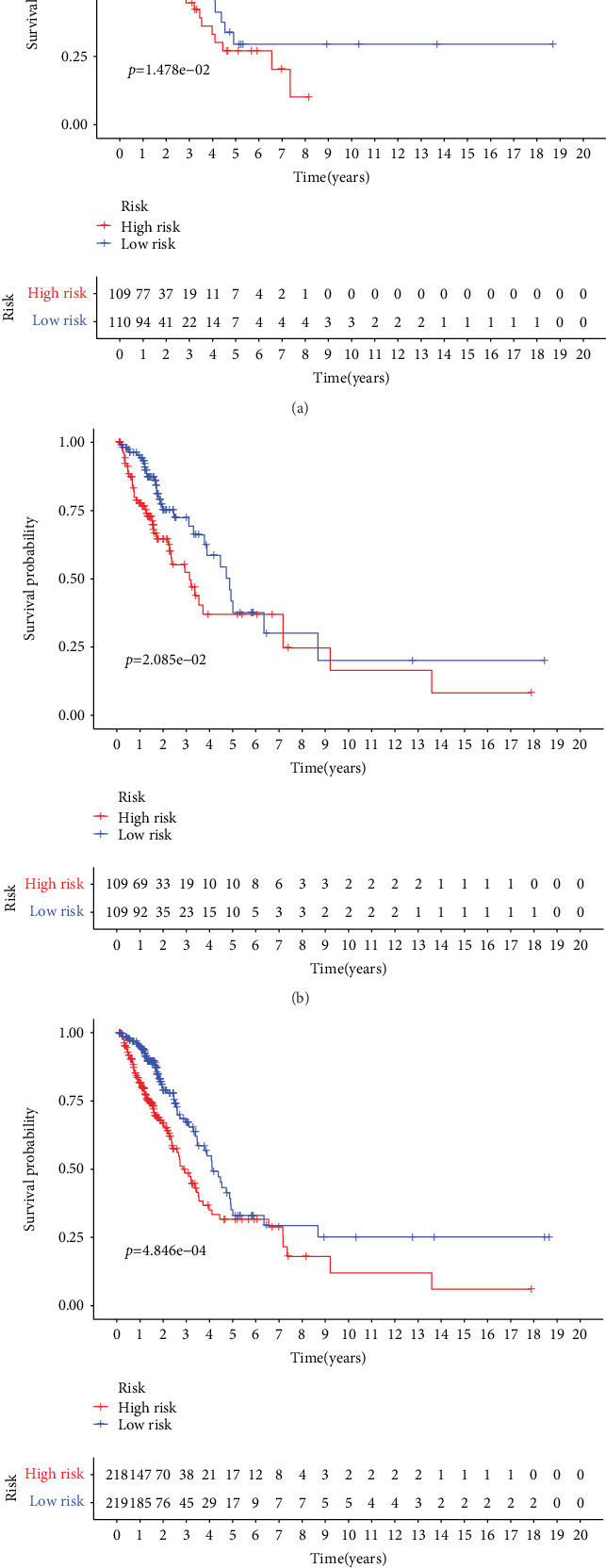
Kaplan–Meier curves of the risk signature in three sets. (a) Training set. (b) Validation set. (c) Combination set.

**Figure 4:**
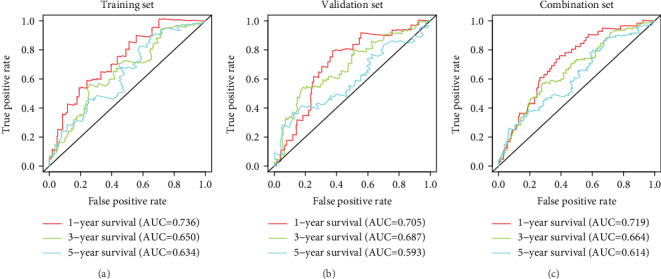
ROC curves of 1-, 3-, and 5-year overall survivals of the risk signature in three sets. (a) Training set. (b) Validation set. (c) Combination set. ROC: receiver operating characteristic.

**Figure 5:**
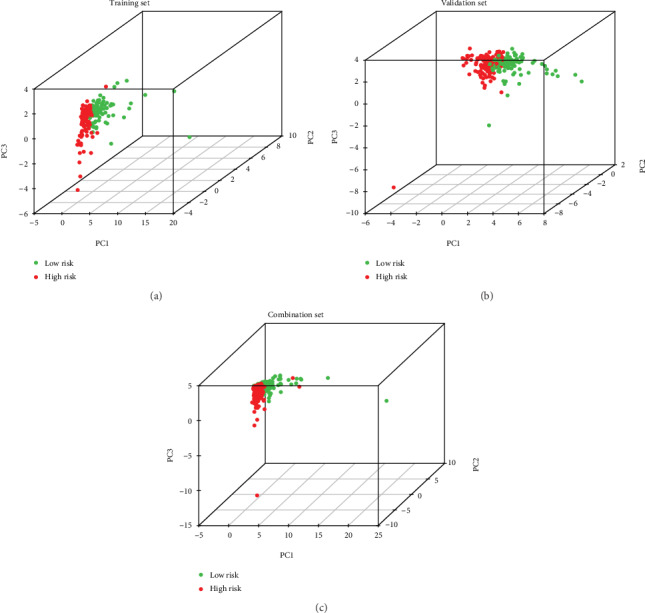
Principal component analysis (PCA) based on the seven immune-related lncRNAs showed that the low-risk group and high-risk group tended to separate into two sides. (a) Training set. (b) Validation set. (c) Combination set.

**Figure 6:**
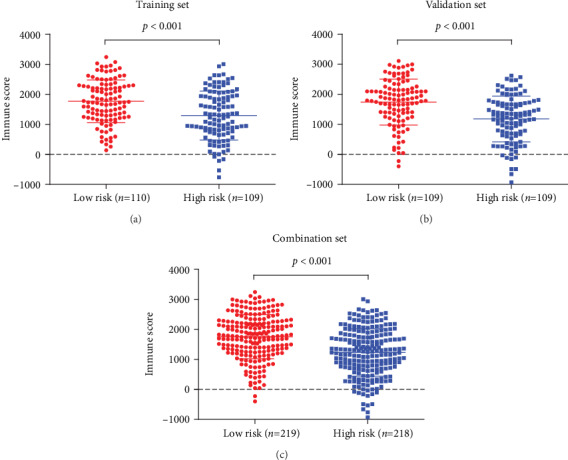
The immune scores in the low-risk group were significantly higher than those in the high-risk group in the training, validation, and combination sets. (a) Training set. (b) Validation set. (c) Combination set.

Univariate Cox regression analysis of the immune-related lncRNAs in the training set showed that seven immune-related lncRNAs (AC092794.1, AL034397.3, AC069023.1, AP000695.1, AC091057.1, HLA-DQB1-AS1, and HSPC324) had the most significant prognostic value. AC092794.1, AP000695.1, and AC091057.1 were risk factors, and AL034397.3, AC069023.1, HLA-DQB1-AS1, and HSPC324 were protective factors. In the LASSO Cox regression algorithm, all of them were identified to construct the risk signature (Figures 2(a) and 2(b)). The coefficients are shown in Table 2. The risk score of each sample was calculated by the sum of the coefficients of each lncRNA multiplied by the corresponding expression in each sample. Based on the median risk score of -0.04074, the training set was divided into low-risk and high-risk groups (Figure 2(c)). Patients in the low-risk group had a longer OS than patients in the high-risk group (*p* = 1.478*e* − 02, Figure 3(a)). AUCs in the training set for 1-, 3-, and 5-year OSs were 0.736, 0.650, and 0.634, respectively (Figure 4(a)), indicating that the risk signature could predict the 1-year survival rates for the LUAD patients better than the 3- and 5-year OS rates. PCA of the low-risk and high-risk groups showed that they could be separated based on the seven immune-related lncRNAs (Figure 5(a)). That is to say, immune-related lncRNAs were used to separate the LUAD patients into two sections, indicating that the immune status of the LUAD patients in the low-risk group was distinguishable from that in the high-risk group. ESTIMATE analysis showed that the immune scores in the low-risk group ranged from 135.4115 to 3241.226, and those in the high-risk group ranged from -766.567 to 3002.727. The immune scores in the low-risk group were significantly higher than those in the high-risk group (Figure 6(a), *p* < 0.001), suggesting that the tumor cells in the low-risk group had more immune cell infiltration than those in the high-risk group.

### 3.3. Validation of the Risk Signature

To verify the accuracy of the risk signature model built by the training set, the seven coefficients were applied to the validation set and the combination set to confirm the risk score of each sample in them. Then, the same analyses used for the training set were used for the validation set and the combination set (Figures 2(d) and 2(e)). Both sets showed results similar to those of the training set. Patients in the low-risk group had longer OS rates than those in the high-risk group (*p* = 2.085*e* − 02 in the validation set and *p* = 4.846*e* − 04 in the combination set, Figures 3(b) and 3(c)). The ROC curves showed that the AUCs for the 1-, 3-, and 5-year OS rates in the validation set were 0.705, 0.687, and 0.593, respectively, and in the combination set, they were 0.719, 0.664, and 0.614, respectively (Figures 4(b) and 4(c)), indicating that the risk signature in both sets could predict the 1-year survival rate for LUAD patients better than the 3- and 5-year OS rates. PCA of both sets showed that the low-risk and high-risk groups were divided into two clusters (Figures 5(b) and 5(c)). ESTIMATE analysis showed that, in the validation set, the immune scores in the low-risk group ranged from -403.82 to 3107.108 and in the high-risk group ranged from -936.191 to 2619.29; in the combination set, the immune scores in the low-risk group ranged from -403.82 to 3241.226 and in the high-risk group ranged from -936.191 to 3002.727. In both of the two sets, the immune scores in the low-risk group were significantly higher than those in the high-risk group (Figures 6(b) and 6(c), *p* < 0.001). These results demonstrated the accuracy and reliability of the risk signature model.

### 3.4. Independent Prognostic Factors

To identify the independent prognostic factors, univariate and multivariate Cox regression analyses were performed in the training set. Age, gender, TNM stage, and risk signature were included. In multivariate Cox analysis, clinicopathological features that were not significant in univariate Cox analysis were excluded. The results indicated that the risk signature and TNM stage were independent prognostic factors. Furthermore, the same results were obtained for the validation set and the combination set, which verified the accuracy of the training set results (Table 3).

### 3.5. Clinicopathological Features in the Low-Risk and High-Risk Groups

Correlation between the clinicopathological features and the risk signature was studied to reveal the distribution of clinicopathological features in the low-risk and high-risk groups (Table 4). In the training set, the survival status was correlated with the risk signature (*p* = 0.041) and the patients in the high-risk group had a poor prognosis. In the validation set, patients with a lower TNM stage were more prevalent in the low-risk group (*p* = 0.008). The combination set had the same results as the training set and the validation set (TNM stage: *p* = 0.005, survival status: *p* = 0.004). In addition, males in the combination set were more likely to be in the high-risk group (*p* = 0.024).

### 3.6. Functional Enrichment Analysis

**Figure 7:**
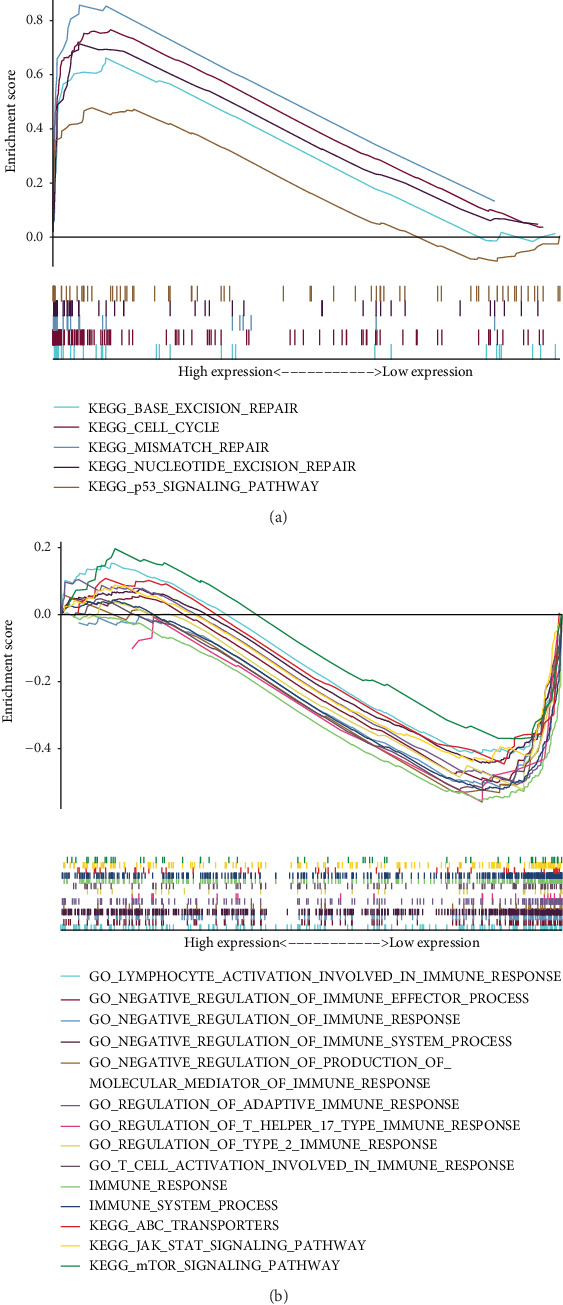
Representative Gene Set Enrichment Analysis (GSEA) showed potential Kyoto Encyclopedia of Genes and Genomes (KEGG) pathways and Gene Ontology (GO) pathways in the low- and high-risk groups. (a) High-risk group. (b) Low-risk group. ABC: ATP-binding cassette; mTOR: mammalian target of rapamycin.

Gene Set Enrichment Analysis (GSEA) between the low-risk and high-risk groups based on the training set revealed some important pathways involved not only in the occurrence and development of cancer but also in immune-related cancer processes. The potential Kyoto Encyclopedia of Genes and Genomes (KEGG) pathways in the high-risk group were mainly enriched in base excision repair, cell cycle, mismatch repair, nucleotide excision repair, and the p53 signaling pathway. The potential functions in the low-risk group were mainly enriched in ATP-binding cassette (ABC) transporters, the JAK-STAT signaling pathway, and the mTOR signaling pathway. The Gene Ontology (GO) analyses revealed that a large number of immune-related processes were enriched in the low-risk group compared to the high-risk group. In addition, the immune response (*p* = 0.02, FDR = 0.02) and immune system process (*p* = 0.016, FDR = 0.016) pathways were enriched in the low-risk group (Figure 7, Table 5). These results indicate that the high-risk group was more likely to be associated with the malignancy of LUAD and the low-risk group was more likely to be associated with the immune-related processes of LUAD.

### 3.7. Construction and Validation of a Nomogram

**Figure 8:**
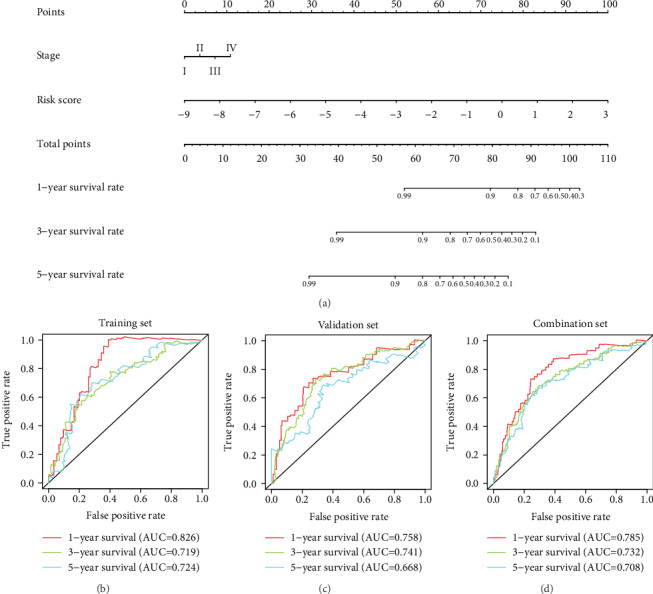
Building and validation of the nomogram predicting overall survival for lung adenocarcinoma patients in the training set. (a) The nomogram built based on the TNM stage and risk signature. (b–d) ROC curves of the training set, validation set, and combination set. ROC: receiver operating characteristic.

**Figure 9:**
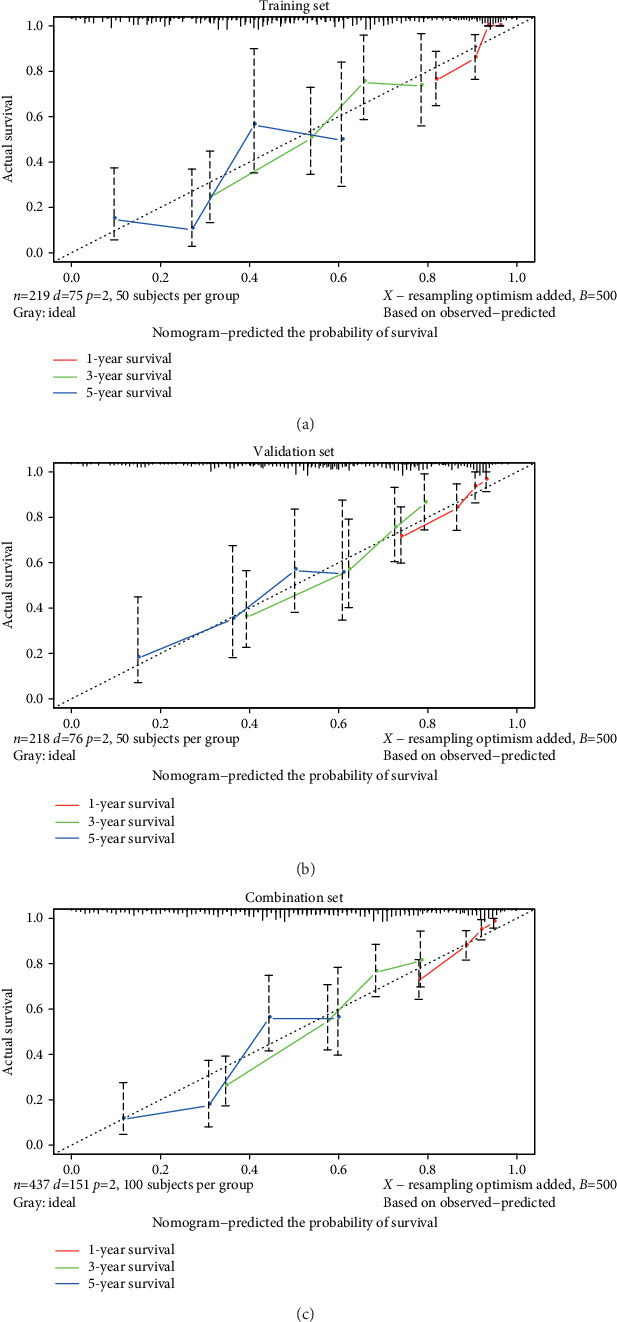
Calibration plots showed that there was good agreement between the actual observations and predictions. (a) Training set. (b) Validation set. (c) Combination set.

A nomogram was built for the training set based on the TNM stage and risk score (Figure 8(c)). The C-index of the nomogram was 0.755, and the AUCs for the 1-, 3-, and 5-year survivals were 0.826, 0.719, and 0.724, respectively (Figure 8(b)). In the validation set and the combination set, the C-indexes were 0.703 and 0.728, respectively. The 1-, 3-, and 5-year AUCs were 0.758, 0.741, and 0.668, respectively, in the validation set (Figure 8(c)) and 0.785, 0.732, and 0.708, respectively, in the combination set (Figure 8(d)). The calibration plots for the 1-, 3-, and 5-year survivals indicated good agreement between the actual observations and the predictions, not only in the training set but also in the validation and combination sets (Figure 9). These results indicated that the prediction performance of the nomogram was good.

## 4. Discussion

In the present study, we systematically collected data from TCGA dataset and extracted immune-related lncRNAs. Then, we identified seven prognostic immune-related lncRNAs through univariate Cox regression analysis and used them to derive a risk signature, which stratified LUAD patients into high- and low-risk categories. Patients in the low-risk group had longer OS than patients in the high-risk group. The AUCs showed that the risk signature had a good predictive value for 1-year survival, which was confirmed by the validation set and the combination set. Finally, a nomogram was built based on age, gender, TNM stage, and risk score, and the prediction performance was good not only in the training set but also in the validation and combination sets.

Seven immune-related lncRNAs played important roles in our study, and a risk signature was constructed based on them. The risk signature was strongly correlated with the OS of LUAD patients and could also predict 1-year survival, not only in the training set but also in the validation and combination sets. The distribution of the TNM stage was different between the low- and high-risk groups in at least two sets. There were more patients with higher TNM stages (III+IV) in the high-risk group, which led to poor prognoses, indicating that the risk signature and TNM stage were crucial prognostic factors. Besides, univariate and multivariate Cox regression analyses revealed that the TNM stage and risk signature were independent prognostic factors. The distribution of gender was different only in the combination set, in which the high-risk group had more male patients. The risk signature was a reliable prognostic model with potential clinical significance. In clinical work, to predict the prognosis of patients, we only need the expression of the seven immune-related lncRNAs; then, risk scores can be calculated based on the coefficients, determining whether the patients are classified as low or high risk to predict their prognoses. With the development of gene sequencing technology, it will soon become a reality. The seven immune-related lncRNAs were novel biomarkers of LUAD and had important prognostic significance. They may become new targets for immunotherapy and lead to new therapeutic strategies. Unfortunately, there have been no prior reports on them. However, this opens up many avenues of study to pursue on this topic.

Furthermore, the risk signature was included in the construction of a nomogram. Compared to the risk signature, a nomogram can include more factors that impact the prognosis and can comprehensively evaluate the prognosis of patients with more accurate results. However, a simple nomogram only contains clinicopathological features. The addition of the risk signature made it more reliable because the prognosis prediction depended not only on the clinicopathological features but also on the expression of related genes, making the results more accurate. Our nomogram showed that higher TNM stages and risk scores were correlated with higher points, and a higher total score was significantly correlated with worse prognoses. The validation and combination sets confirmed the accuracy and reliability of the nomogram.

The functional enrichment analysis suggested that our risk signature was related not only to immune function but also to the malignancy of LUAD. p53 plays a crucial role in the cell cycle, apoptosis, and genomic stability (22, 23). Studies have reported that p53 mutations not only caused a loss of anticancer function but also acquired the process of carcinogenesis, which would lead to the migration, invasion, and metastasis of early cancer (24, 25). High expression of ATP-binding cassette (ABC) transporters could reduce the concentration of cisplatin in tumor cells and lead to cisplatin resistance in lung cancer (26, 27). Julian et al. reported that the JAK-STAT signaling pathway was associated with the progression of LUAD (28). In addition, JAK signaling was involved in the formation of TME by regulating T cell, natural killer (NK), and dendritic cell function (29). Evidence has demonstrated that the mammalian target of rapamycin (mTOR) signaling pathway was associated with metastasis and cisplatin resistance in lung adenocarcinoma (30, 31). GO analyses also suggested that the low-risk group was associated with immune-related processes, some of which were negatively regulated, and the results of ESTIMATE analysis showed that the tumor cells in the low-risk group had more immune cell infiltration than those in the high-risk group. These results indicate that our risk signature was strongly correlated with the immune function and malignancy of LUAD.

There were limitations to this study. (1) We tried to use the datasets in the Gene Expression Omnibus (GEO) database (https://www.ncbi.nlm.nih.gov/geo/) as the validation set, but due to the sequencing method, the number of lncRNAs in the GEO datasets was so small that they could not be used as the validation set. Therefore, we could only randomly and equally divide TCGA dataset into the training set and the validation set, which inevitably increased the bias in the study. (2) Due to incomplete clinicopathological features, fewer clinicopathological features, such as survival time, survival status, age, gender, and TNM stage, could be used. (3) The functions of the seven lncRNAs have not been validated at present, so more experimental data are needed to support our findings. (4) The number of corresponding adjacent LUAD or normal tissues and the number of LUAD tissues are extremely unbalanced in TCGA, so differential analysis of the seven lncRNAs between normal tissues and tumor tissues is unavailable.

## 5. Conclusion

In conclusion, we identified seven immune-related lncRNAs as potential biomarkers of LUAD. This was the first study to generate a risk signature based on the immune-related lncRNAs of LUAD. A nomogram was also built that included the patient clinicopathological features and risk signature, which could predict the 1-, 3-, and 5-year survival rates of LUAD patients. Our study not only has important significance in predicting the prognosis of LUAD but may also guide future immunotherapy.

## Figures and Tables

**Table 1:** Clinicopathological features of lung adenocarcinoma patients in the training set and validation set.

Characteristics	Training set (*n* = 219)	Validation set (*n* = 218)	*p* value
Age			0.364
≤65	102	111	
>65	117	107	
Gender			0.468
Male	102	94	
Female	117	124	
Survival status			0.892
Alive	144	142	
Dead	75	76	
TNM stage			0.36
I+II	169	160	
III+IV	50	58	

**Table 2:** Seven prognostic immune-related lncRNAs identified from Pearson's correlation analysis and univariate Cox regression analysis.

Gene symbol	Ensembl ID	Coefficient	Univariate Cox regression analysis			
			HR	95% CI lower	95% CI higher	*p* value
AC092794.1	ENSG00000274987.1	0.091612549	1.119	1.004	1.248	0.042
AL034397.3	ENSG00000274536.7	-0.215357523	0.653	0.454	0.939	0.021
AC069023.1	ENSG00000235637.1	-0.131327244	0.016	0.000	0.824	0.040
AP000695.1	ENSG00000230479.1	0.277874381	1.413	1.116	1.789	0.004
AC091057.1	ENSG00000187951.11	0.350748081	1.523	1.054	2.201	0.025
HLA-DQB1-AS1	ENSG00000223534.1	-0.037782602	0.925	0.863	0.991	0.027
HSPC324	ENSG00000228401.4	-0.288418544	0.483	0.260	0.899	0.022

**Table 3:** Univariate and multivariate Cox analyses of the risk signature and clinicopathological features for the independent prognostic value in lung adenocarcinoma patients.

	Variables	Univariate Cox regression analysis			
		HR	95% CI lower	95% CI higher	*p* value
Training set	Age	1.015	0.991	1.039	0.224
	Gender	0.959	0.609	1.511	0.857
	TNM stage	1.625	1.331	1.984	<0.001
	Risk signature	2.841	1.895	4.258	<0.001
Validation set	Age	1.004	0.981	1.028	0.712
	Gender	1.213	0.771	1.908	0.405
	TNM stage	1.734	1.395	2.155	<0.001
	Risk signature	1.765	1.217	2.561	0.003
Combination set	Age	1.010	0.993	1.027	0.244
	Gender	1.054	0.765	1.452	0.748
	TNM stage	1.689	1.460	1.955	<0.001
	Risk signature	2.215	1.706	2.876	<0.001
	Variables	Multivariate Cox regression analysis			
		HR	95% CI lower	95% CI higher	*p* value
Training set	TNM stage	1.870	1.160	3.015	0.01
	Risk signature	2.540	1.653	3.904	<0.001
Validation set	TNM stage	2.664	1.622	4.376	<0.001
	Risk signature	1.539	1.075	2.204	0.019
Combination set	TNM stage	2.274	1.611	3.210	<0.001
	Risk signature	1.922	1.469	2.515	<0.001

HR: hazards ratio; CI: confidence interval.

**Table 4:** Correlation of the clinicopathological features of lung adenocarcinoma patients and risk signature in this study.

Characteristics	Training set (*n* = 219)			Validation set (*n* = 218)			Combination set (*n* = 437)		
	Low risk (*n* = 110)	High risk (*n* = 109)	*p* value	Low risk (*n* = 109)	High risk (*n* = 109)	*p* value	Low risk (*n* = 219)	High risk (*n* = 218)	*p* value
Age			0.312			1			0.668
≤65	47	55		56	55		104	109	
>65	63	54		53	54		115	109	
Gender			0.120			0.132			0.024
Male	45	57		41	53		86	110	
Female	65	52		68	56		133	108	
Survival status			0.041			0.065			0.004
Alive	80	64		78	64		158	128	
Dead	30	45		31	45		61	90	
TNM stage			0.137			0.008			0.005
I+II	90	79		94	66		183	157	
III+IV	20	30		15	43		36	61	

**Table 5:** Representative results of Gene Set Enrichment Analysis.

Group	Name	Size	NES	NOM *p* value	FDR *q* value
High-risk group	KEGG_BASE_EXCISION_REPAIR	35	1.901	0.013	0.025
	KEGG_CELL_CYCLE	124	2.381	<0.001	<0.001
	KEGG_MISMATCH_REPAIR	23	2.091	<0.001	0.005
	KEGG_NUCLEOTIDE_EXCISION_REPAIR	44	2.139	<0.001	0.003
	KEGG_p53_SIGNALING_PATHWAY	68	1.787	0.006	0.045

Low-risk group	KEGG_ABC_TRANSPORTERS	44	-1.647	0.014	0.111
	KEGG_JAK_STAT_SIGNALING_PATHWAY	155	-1.783	0.012	0.060
	KEGG_mTOR_SIGNALING_PATHWAY	52	-1.528	0.020	0.160
	GO_NEGATIVE_REGULATION_OF_IMMUNE_RESPONSE	140	-1.920	0.002	0.068
	GO_NEGATIVE_REGULATION_OF_IMMUNE_EFFECTOR_PROCESS	118	-1.920	0.004	0.067
	GO_REGULATION_OF_ADAPTIVE_IMMUNE_RESPONSE	160	-1.860	0.010	0.076
	GO_NEGATIVE_REGULATION_OF_IMMUNE_SYSTEM_PROCESS	447	-1.850	0.002	0.075
	GO_T_CELL_ACTIVATION_INVOLVED_IN_IMMUNE_RESPONSE	99	-1.840	0.013	0.072
	GO_REGULATION_OF_TYPE_2_IMMUNE_RESPONSE	30	-1.780	0.004	0.084
	GO_NEGATIVE_REGULATION_OF_PRODUCTION_OF_MOLECULAR_MEDIATOR_OF_IMMUNE_RESPONSE	36	-1.760	0.008	0.089
	GO_REGULATION_OF_T_HELPER_17_TYPE_IMMUNE_RESPONSE	19	-1.700	0.027	0.105
	GO_LYMPHOCYTE_ACTIVATION_INVOLVED_IN_IMMUNE_RESPONSE	175	-1.620	0.045	0.125
	IMMUNE_RESPONSE	234	-1.836	0.020	0.020
	IMMUNE_SYSTEM_PROCESS	331	-1.914	0.016	0.016

NES: normalized enrichment score; FDR: false discovery rate; KEGG: Kyoto Encyclopedia of Genes and Genomes; GO: Gene Ontology; ABC: ATP-binding cassette; mTOR: mammalian target of rapamycin.

## Data Availability

The datasets for this study can be found in the TCGA database (https://portal.gdc.cancer.gov/) and Molecular Signatures Database (http://www.gencodegenes.org).
